# Systemic effects of oral tolerance in bone healing

**DOI:** 10.1038/s41598-023-33591-4

**Published:** 2023-04-18

**Authors:** Bruno Henrique Costa, Alisson Kennedy Rezende, Lais Costa, Gabrielle Fernanda Monteiro Neves, Antônio Carlos Shimano, Álvaro de Oliveira Penoni, Claudia Rocha Carvalho, Raquel Alves Costa, Erika Costa de Alvarenga

**Affiliations:** 1 Departamento de Ciências Naturais, Universidade Federal de São João Del Rei, Praça Dom Helvécio 74, São João del Rei, Minas Gerais 36301-160 Brazil; 2 Departamento de Biomecânica, Medicina e Reabilitação do Aparelho Locomotor, Faculdade de Medicina de Ribeirão Preto, Universidade de São Paulo, Ribeirão Preto, São Paulo 14049-900 Brazil; 3 Departamento Morfologia, Instituto de Ciências Biológicas, Universidade Federal de Minas Gerais, Belo Horizonte, Minas Gerais 31270-901 Brazil; 4 Departamento das Ciências da Educação Física e Saúde, Universidade Federal de São João del-Rei, São João del Rei, Minas Gerais 36301-360 Brazil

**Keywords:** Musculoskeletal system, Physiology, Bone, Bone quality and biomechanics, Immunology, Applied immunology, Inflammation, Osteoimmunology, Vaccines

## Abstract

Bone fractures cause acute inflammation that, despite being important for initial repair, may delay the healing of the damaged bone. Parenteral injection of dietary protein has been shown to decrease inflammation and accelerate the repair of skin wounds and other inflammatory pathologies. Thus, our aim was to evaluate whether the intraperitoneal (i.p.) immunization with zein, an abundant protein in rodent chow, would favor bone healing. Wistar rats received i.p. immunization: saline (SG), adjuvant (AG) and zein associated with adjuvant (ZG). Then, a 2 mm of defect bone was performed on the right tibia, and on days 7, 14, 28 and 45 thereafter, analyses were performed. The results showed that the injection of zein reduced inflammation without impairing bone mineralization. Moreover, biomechanical tests demonstrated higher levels of maximum force (N) in ZG, indicating better mechanical resistance in relation to the others. The computerized tomography also indicated lower levels of medullary content in the ZG than in the SG, suggesting the absence of trabeculae in the medullary region in the ZG. These findings suggest that the injection of zein in previously tolerated animals may improve bone repair, leading to mechanically functional bone formation.

## Introduction

Life expectancy has gradually increased worldwide with a consequent surge of bone fracture chances. Bone fractures induced by various causes are common around the world and are followed by pain, disability, and reduced mobility, which decrease the quality of life. In healthy individuals, a normal healing bone process occurs without further complications. However, in cases with larger bone defects or other concomitant pathologies, healing can be affected, resulting in longer recovery periods or even nonunion. Additionally, some fractures related to osteoporosis may result in death. One in three women and at least one in six men will suffer an osteoporotic fracture in their lifetime since the life expectancy is 73 years old in the world^1^. It is estimated that more than 23 million people are at high risk of osteoporotic fractures in the European Union (EU), and healthcare system expenses exceed €56 billion each year^2^.

Bone tissue, unlike other tissues, repairs itself without forming a scar. However, the fracture repair process will occur depending on the type of fracture, which can be noncritical or critical (large-area injuries). Bone repair is divided into overlapping phases: hemostasis, inflammation, proliferation and remodeling. During homeostasis, the hematoma is formed by factors released by blood platelets and fibrin at the site of injury. In addition, proinflammatory cytokines and growth factors are also released, such as tumor necrosis factor-alpha (TNF-α), interleukin-1 (IL-1), IL-6, IL-11 and IL-18^3,4^. The inflammation starts with an increased angiogenesis at the site of injury, followed by infiltration of circulating neutrophils and macrophages in addition to resident macrophages (M1). These cells release pro-inflammatory cytokines, which activate the adaptive immune response (T Lymphocytes) and promote the migration of MSC (Mesenchymal Stem Cells) to the hematoma in osteogenic cells. In the subsequent proliferative phase, macrophages acquire mostly an anti-inflammatory and angiogenic phenotype (M2) in response to a change in the surrounding cellular and cytokine^5^. This process is then followed by an influx of fibroblasts, establishing the proliferation phase. In the peripheral regions of the fracture, intramembranous ossification occurs 7–10 days after injury. In the inner part of the fracture, the granulation tissue is replaced by fibrous tissue and fibrocartilage and later soft callus cartilage. This is later transformed into a hard callus, which is mineralized and a matrix produced by osteoblasts^6,7^. This phase transition is dependent on the cure of the inflammation, and wounds are at risk of becoming chronic when inflammation persists^8^.

Thus, a successful inflammatory phase transition is essential for bone fracture repair. A biological phenomenon that demonstrates a systemic reduction in inflammation in various experimental inflammatory models is the indirect effect of oral tolerance. Oral tolerance is an immunological phenomenon initiated in the gut-associated lymphoid tissue (GALT) that blocks the initiation of an immune response to feed antigens. Oral tolerance also reduces inflammation in many experimental inflammatory models^9^. Despite the vast majority of the proteins ingested as food being degraded within the gastrointestinal tract, antigenically intact molecules can still be absorbed in sufficient amounts to modulate immune responses. Thus, when a protein is contacted by oral route, the immune cells are activated, before parenteral immunization with this same protein, and oral tolerance is established^10,11^. Oral tolerance requires antigen uptake into the GALT followed by dendritic cell (DC) migration to the mesenteric lymph node and induction of gut-homing imprinted regulatory T cells (Treg cells)^10,12^.

Besides the specific nature of oral tolerance, parenteral injection of tolerated proteins triggers systemic effects, a phenomenon sometimes referred to as bystander suppression^9,13^. In this way, systemic effects of oral tolerance inhibit inflammatory responses to many agents injected concomitantly with the tolerated protein^14,15^. Thus, the systemic effects triggered by parenteral exposure to protein contacted by the oral route are not limited to the intestinal mucosa. Previous works demonstrated that parenteral reexposure to tolerated proteins such as ovalbumin (OVA) or zein concomitant with skin wounds reduces the inflammatory phase, leading to an improvement in tissue repair^14–17^ Cantaruti et al.^14^ showed that intraperitoneal immunization with zein minutes before skin wounding in mice reduces the inflammatory infiltrate and improves wound healing.

As systemic effects of oral tolerance improve repair, for example in skin, we aimed to evaluate if they could also affect bone repair. Several treatments for bone repair are available, but many of them will interfere with the inflammatory phase, triggering side effects in patients. Other approaches will require a new material or medicine that is not always economically accessible, and the extensive time spent on bone repair displays an economic impact on the global health system. Thus, the development of a low-cost treatment that is minimally invasive and capable of improving bone repair will benefit millions of people. Parenteral injection of a tolerated protein from the diet exhibits systemic effects in several inflammatory models already described in the literature. Considering the crucial role of inflammation in bone repair and the inhibitory inflammatory response by systemic oral tolerance, we examined whether the injection of zein concomitant with a bone defect in rat tibia reduces the inflammatory infiltrate and promotes successful bone healing.

## Material and methods

### Animals

**Figure 1:**
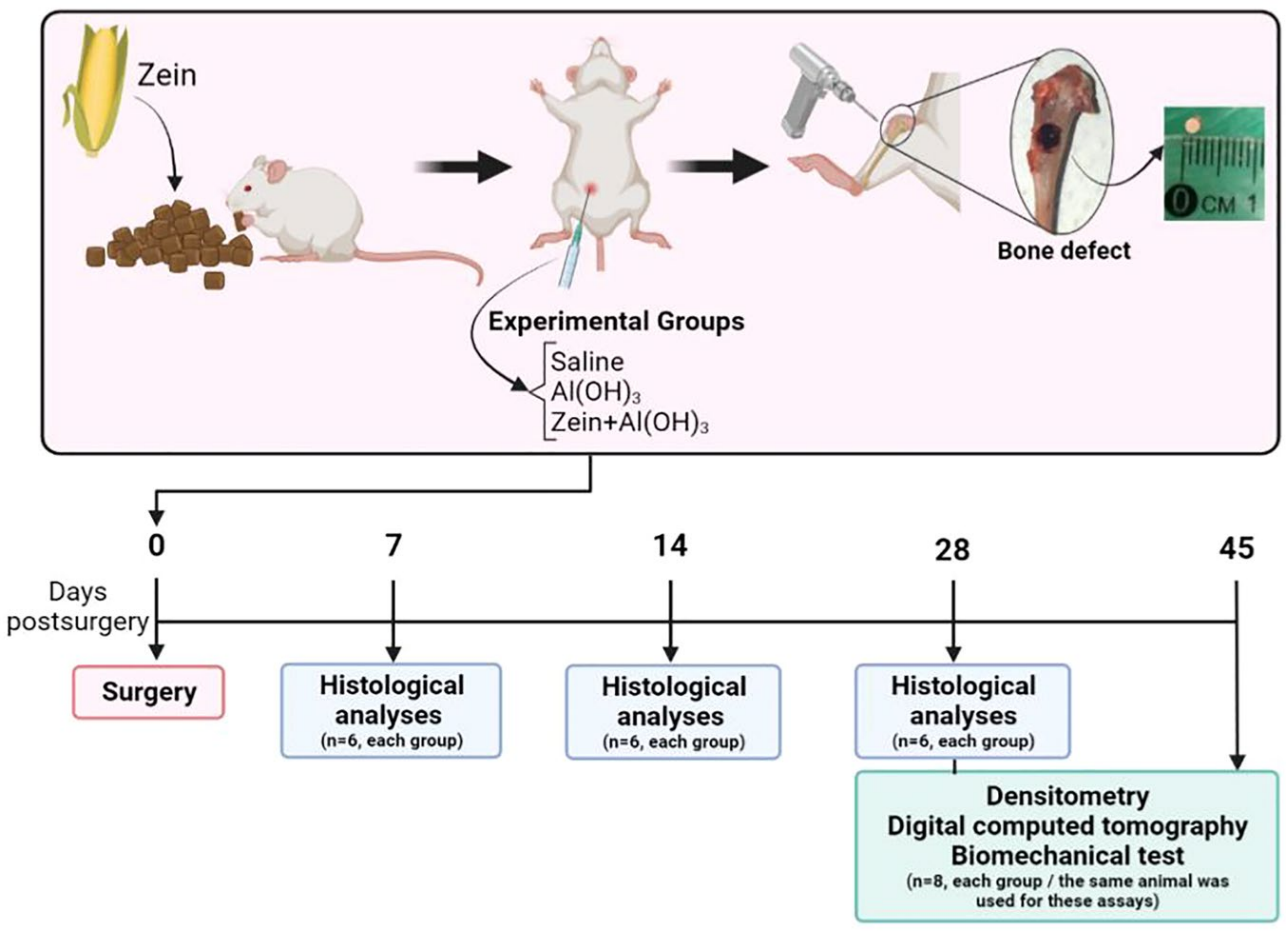
Experimental summary. Rats were fed a zein-enriched diet. At twelve weeks of age, each rat underwent surgery to create a circular bone defect of 2 mm in diameter in the proximal middle third of the right tibia. Around 5 min before surgery, an intraperitoneal injection containing either saline, Al(OH)_3_ adjuvant, or zein protein with Al(OH)_3_ was applied for immunization. The animals were euthanized at the indicated experimental dates. After 7, 14 and 28 days after surgery, the bones underwent histological analysis. After 28 and 45 days, the defective bones were evaluated by mineral densitometry, digital computed tomography and biomechanical testing.

Twelve-week-old male Wistar rats were provided by the animal breeding center of the Universidade Federal de São João del Rei (UFSJ), Brazil, and treated according to the National Council for Control of Animal Experimentation (CONCEA) standards and approved by the Ethics Committee of Animal Experimentation of UFSJ under protocol number 054/2017. This study is reported in accordance with ARRIVE guidelines (https://arriveguidelines.org). The animals received feed and water ad libitum. Tolerance was induced through a feed that already contained zein protein in its composition (Nuvilab CR- 1, Nuvital Nutrientes S/A, São Paulo, Brazil). The groups contained six or eight rats per time point, according to the specific assay (Fig. 1).

### Parenteral injection of dietary protein

Purified zein (Sigma, St Louis, MO) was used for intraperitoneal immunization minutes before the surgery. The animals were divided into 3 distinct groups. The zein group (ZG) was immunized with 0.5 ml of 60 µg zein plus 9.6 mg of Al(OH)_3_. One control group adjuvant (AG) received 9.6 mg of Al(OH)_3,_ and the other control group received saline intraperitoneally (SG). The experimental analyses were 7, 14, 28, and 45 days after the surgical bone defect was processed. For this study, we used N = 6 for histological analysis and N = 8 for the biomechanical test (Fig. 1).

### Bone defect model

To perform this surgery, the animals were anesthetized with a combination of xylazine (0.15 mg/animal) and ketamine (0.30 mg/animal). Then, the surgical area was trichotomized and disinfected. One noncritical bone defect was surgically created by using a motorized drill (with a 2 mm diameter) under copious irrigation with saline solution at the upper third of the right tibia. The skin was sutured, and asepsis was performed with povidone-iodine 10%^18^. After surgery, the animals received a subcutaneous injection of the antibiotic enrofloxacin (0.2 ml/animal diluted in saline) once. The animals received oral enrofloxacin (0.025 mg/ml) and dipyrone sodium (25 mg/animal/day) for three consecutive days after the surgery. Rats were euthanized individually at experimental times of 07, 14, 28, and 45 days post-surgery, and the tibias were removed for subsequent analysis. These specific days were defined according to Einhorn^19^ and Prado et al.^18^ because of the possibility to identify chondrogenesis and inflammatory response 7 days after the surgery, cartilaginous callus and periosteal bone formation 14 days after, and calcified cartilage and newly formed woven bone 28 and 45 days after.

### Biomechanical test

This assay was performed according to Yanagihara et al.^20^. The injured tibia was dissected, wrapped in gauze with saline solution, and frozen in a freezer at − 20 °C until the date of the mechanical tests. Twelve hours before the tests, the bones were removed from the − 20° freezer and placed in the refrigerator (1.7° to 3.3°) for natural thawing. Afterwards, they were submerged in saline solution to maintain hydration. The Universal Test Machine (EMIC®, Model DL10.000, Brazil) was used with a load cell with a capacity of 500 N. The device was connected to the Tesc® software (version 13.0, EMIC®, Brazil) used to generate a load versus displacement plot for each test and obtain bone mechanical properties of maximum strength and relative stiffness. To perform the procedure, the distal end of the tibias was embedded in polymethylmethacrylate acrylic resin using powdered methyl ethyl methacrylate polymer (AutoCril®, Brazil) and self-curing liquid methyl methacrylate monomer (Jet®, Brazil). The acrylic resin support was used to protect the distal region of the bones when fixed in the vise. The tibia was fixed at the distal end in a vise, and the force was applied at the proximal end, 8 mm away from the center of the defect. The support point was positioned in the central region of the defect on the opposite side so that the defect was facing upwards, characterizing a 3-point flexion test. The force was applied to the proximal end of the tibia in the mediolateral direction at a speed of 5 mm/min so that the external surface of the defect was subjected to a traction load. The settling time was 30 s with a preload of 10 N^21^.

### Bone mineral density

Bone mineral density (BMD) was assessed by dual-energy X-ray absorptiometry (DXA) using a Lunar DPX-IQ densitometer (Lunar—software version 4.7e, GE Healthcare, UK) designed for small animals and set at high resolution. The tibia was positioned with the bone defect upward and immersed 2 cm deep in saline solution in a plastic container. To stabilize the positioning during the examination, the tibiae were fixed with orthodontic wax at their distal end. Thus, the X-ray incidence was in the medial–lateral direction. With the aid of the same computer program used to acquire the images, the exams were analyzed. Using the manual selection tool, the bones were demarcated in the region of interest of the object (ROI) with an area of 0.074 cm^2^, collecting information about bone mineral content (BMC) and bone mineral density (BMD). To position the ROI over the defect, the protocol was adopted in which the defect site was measured with the aid of a pachymeter using the radiographic image of the bone. The coefficient of variation was 4.5^22^.

### Digital computed tomography

All images were acquired in a Cone-Beam Computed Tomography (CBCT) scanner model Orthophos SL (Siemens, Germany) with the same protocol of 0.08 mm (80.0 µm) voxel (spatial resolution), FOV (field of view) of 5.0 × 5.0 cm, 85 kV and 7 Ma. The tibia was positioned on one support and centered in the FOV using guide light lines. For the analyses, the guidelines were adjusted for each tibia individually so that the sagittal plane coincided with the long axis of the species in question, as well as the coronal plane. The images were analyzed by OnDemand3D software (Cybermed, Seoul, South Korea). Each image was positioned at the defect site, allowing visualization of the cortex of the defect site, the underlying medullary, and the cortex on the opposite side of the defect. This entire procedure was carried out for each tibia of each group, and the maximum and minimum values of the voxel intensity of each segment were tabulated^23^.

### Histological processing

The samples were fixed with Millong's buffered formalin for 48 h. After we realized the decalcification process using 10% Ethylenediaminetetraacetic acid (EDTA—Synth®) in PBS at pH 7.2 for a period of 52 days, it was changed every 3 days. Subsequently, sections were made for histological processing. The bones were divided into 2 halves by sectioning the center of the lesion in the transverse plane, and the separate pieces were washed in running water for 1 h to effectively remove the EDTA, which was dehydrated in ethanol and then embedded in paraffin for histological studies. The paraffin blocks were sectioned in serial transverse sections of 5 μm starting from the middle of the lesion toward the opposite side. The samples were stained with hematoxylin and eosin (H&E).

### Morphometric analysis

Histological bone sections were analyzed under a light optical microscope (Olympus BX51). The images were acquired by the Moticam 2000 system (2.0 M pixel), and the measurements were performed using ImageJ software (https://imagej.nih.gov/ij). Qualitative analyses of bone sections were performed through a score elaborated using the defined parameters in Table 1. The osteocytes were identified by their characteristic morphology in H&E-stained sections: Purple-stained nuclei within gaps located within the pink-stained bone matrix. The connective tissue was identified by a light pink stain with the presence of a large number of nuclei immersed in the collagen fiber matrix. The erythrocyte infiltrate was visualized through stained red regions, in which, with a 40 × objective, it was possible to visualize anucleated cells present in the region. The primary bone tissue was characterized by the presence of irregularly distributed (nonlamellar) osteocytes immersed in the mineralized matrix. Osteocytes were counted within the healing area of the bone defect in five random fields of 32,659 μm^2^ each per animal. Six slides were analyzed, one from each rat, and results were expressed per group as the mean ± SEM. The mean number of osteocytes per field was then calculated by the area analyzed. The area of bone tissue formed in the marrow and cortical region was measured. All qualitative histological description and semiquantitative description (score) were performed double-blind.

**Table 1:** Score developed for qualitative analysis of predetermined parameters related to bone repair.

Score	Bone neoformation	Bone trabeculae	Fibrocartilaginous tissue	Inflammatory infiltrate
−	Absence	Absence	Absence	Absence
+	Minimum	Minimum	Minimum	Minimum
++	Little	Little	Not much	Little
+++	Average	Average	Medium	Medium
++++	High	High	High	High
+++++	Regenerated bone defect	Exacerbated	Exacerbated	Exacerbated

Adapted from: Han et al.^24^ and Sarban et al.^25^.

### Statistical analysis

The analysis of variance and normality tests was performed before starting the experiments. In each experimental phase, we employed three experimental groups with 6 to 8 animals per time point (Fig. 1). This number was estimated by a power analysis in order to ensure the number of viable animals to perform a post-test with more than 10 degrees of freedom after the analysis of variance. To assess the statistical significance of differences between the two groups, we performed two-tailed unpaired Student’s t-tests. To analyze the statistical significance of differences among more than two groups, we performed one-way ANOVAs with Student Newman‒Keuls posttest for multiple comparison tests, with a significance level of 5%. accepted for p ≤ 0.05. Statistical analyses were performed using Prism ver. 8 (GraphPad Software, San Diego, CA, USA).

### Ethical approval

The Animal Care Committee guidelines of the Federal University of São João del Rei (CEUA no. 054/2017) approved the present study, and national guidelines for the care and use of laboratory animals were observed. This study is reported in accordance with ARRIVE guidelines (https://arriveguidelines.org).

## Results

**Figure 2:**
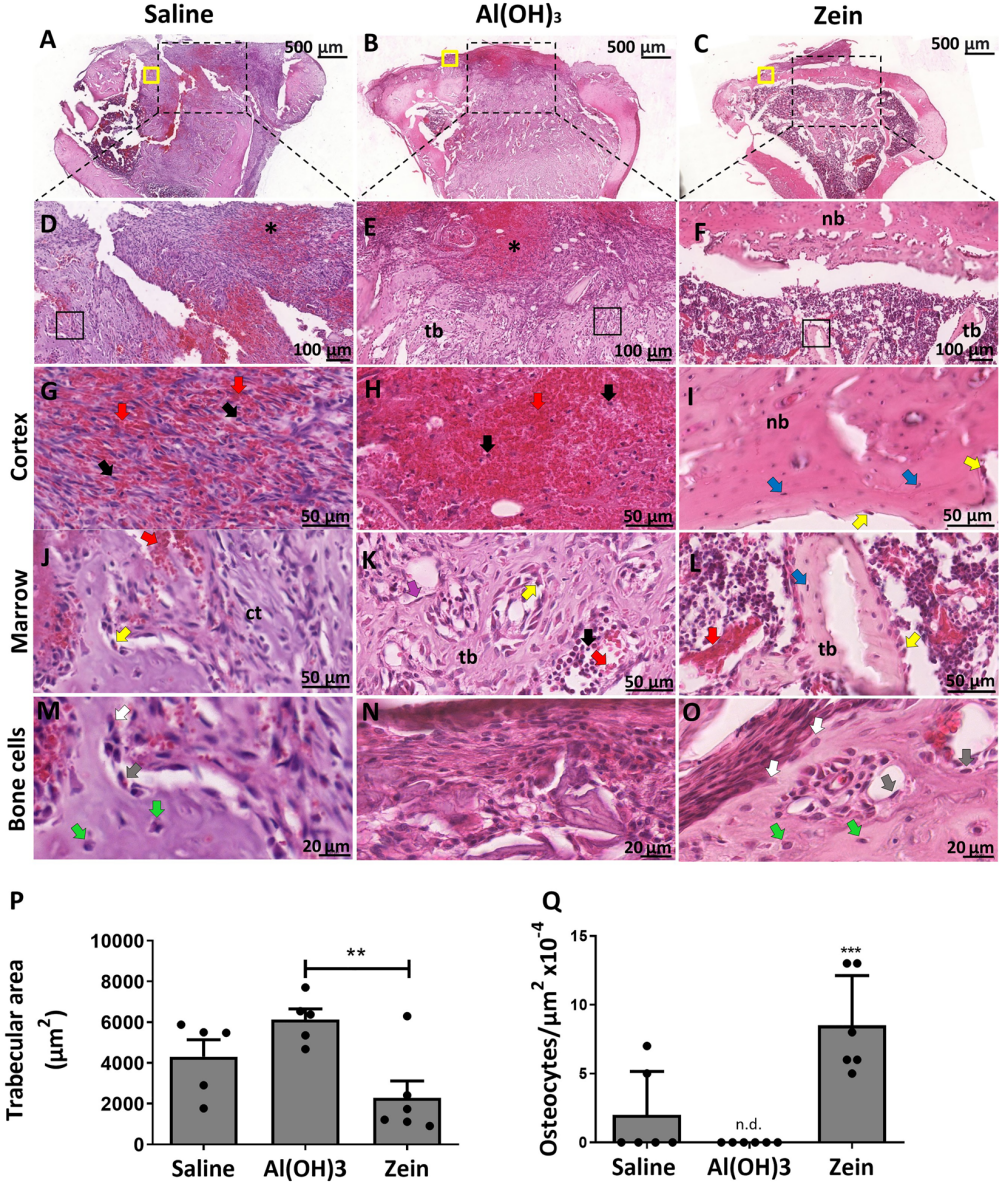
Representative H&E staining images showing the defect region in the rat tibia 7 days post-surgery. (**A**–**C**) Cross-sections of the defect region in the tibia of the studied groups (scale bar = 500 μm, n = 6). (**D**–**F**) Magnification of the defect area (scale bar = 100 μm, n = 6). (**G**–**I**) Cortical defect bone region demonstrating the area of erythrocytes infiltrated with an asterisk (*) and new bone (nb) formation in the Zein group (scale bar = 50 μm, n = 6). (**J**–**L**) The medullary region is delimited by the square with the presence of connective tissue (ct) and trabecular bone (tb), especially in the Zein group (scale bar = 50 μm, n = 6). Red arrows indicate red blood cells, blue arrows indicate osteocytes, yellow arrows indicate osteoblasts, and black arrows indicate the nuclei of various cells immersed in connective tissue. (**M**–**O**) Corresponding to the maturation stages of bone cells. Gray arrows indicate osteoblasts, white arrows indicate osteoblasts in transition to osteocytes and green arrows indicate mature osteocytes. (**P**) Corresponding quantification of the trabecular bone (tb) area in the medullary region. (**Q**) Corresponding quantification of the osteocytes in the cortical region of the defect. Data represent the mean ± SEM, n.d. for not detected, with **p ≤ 0.01 and ***p ≤ 0.001 for statistical analysis performed between the experimental groups, n = 6.

To investigate the hypothesis that intraperitoneal (*i.p*.) immunization with dietary proteins, such as zein, affects the bone healing process, a bone defect model in a rat tibia was performed to evaluate the progress of bone repair (Fig. 1). Then, histological images were analyzed 7 days after the surgery (Fig. 2). The saline group (SG) showed a considerable portion of connective tissue with infiltration of red blood cells and granulation tissue in the cortical region (Fig. 2A,D,G). This feature characterizes the inflammatory stage in bone repair, indicating an initial stage of bone regeneration. In the medullary region, we observed the formation of connective tissue, with cuboidal cells (Fig. 2J) and some bone cells (Fig. 2M). In the defect region, we identified bone neoformation only in the bone extremity in the area of the lesion. The analysis of the adjuvant group (AG) (Fig. 2B) revealed an extensive region marked by infiltration of red blood cells (Fig. 2B,E,H) indicating a persistent hematoma phase. In the cortical defect region no bone neoformation was identified, only connective tissue (Fig. 2N).

Surprisingly, the histological sections of the zein group (ZG) 7 days after the surgery revealed the presence of a new bone matrix and the absence of cartilage in the cortical region of the defect (Fig. 2C,F,I). The presence of osteoblasts was detected in the region of the inner cortical border, and many osteocytes were immersed in the newly formed bone (Fig. 2I,O,Q). In addition, the marrow region in ZG showed a reduced amount of bone trabeculae, where it was possible to observe osteocytes and osteoblasts with morphology characterizing metabolic activity (Fig. 2L,P,Q). In particular, the trabecular bone area in the marrow region was analyzed and expressed in graphs that revealed a greater area in the AG group than in the other groups (Fig. 2M). On day 7 after the bone fracture, there were many osteocytes in the trabecular region of the ZG, and these cells were not observed in the AG (Fig. 2N). These data suggest the tolerized protein (ZG) promoted evident effects in bone repair during the inflammatory stage.

**Figure 3:**
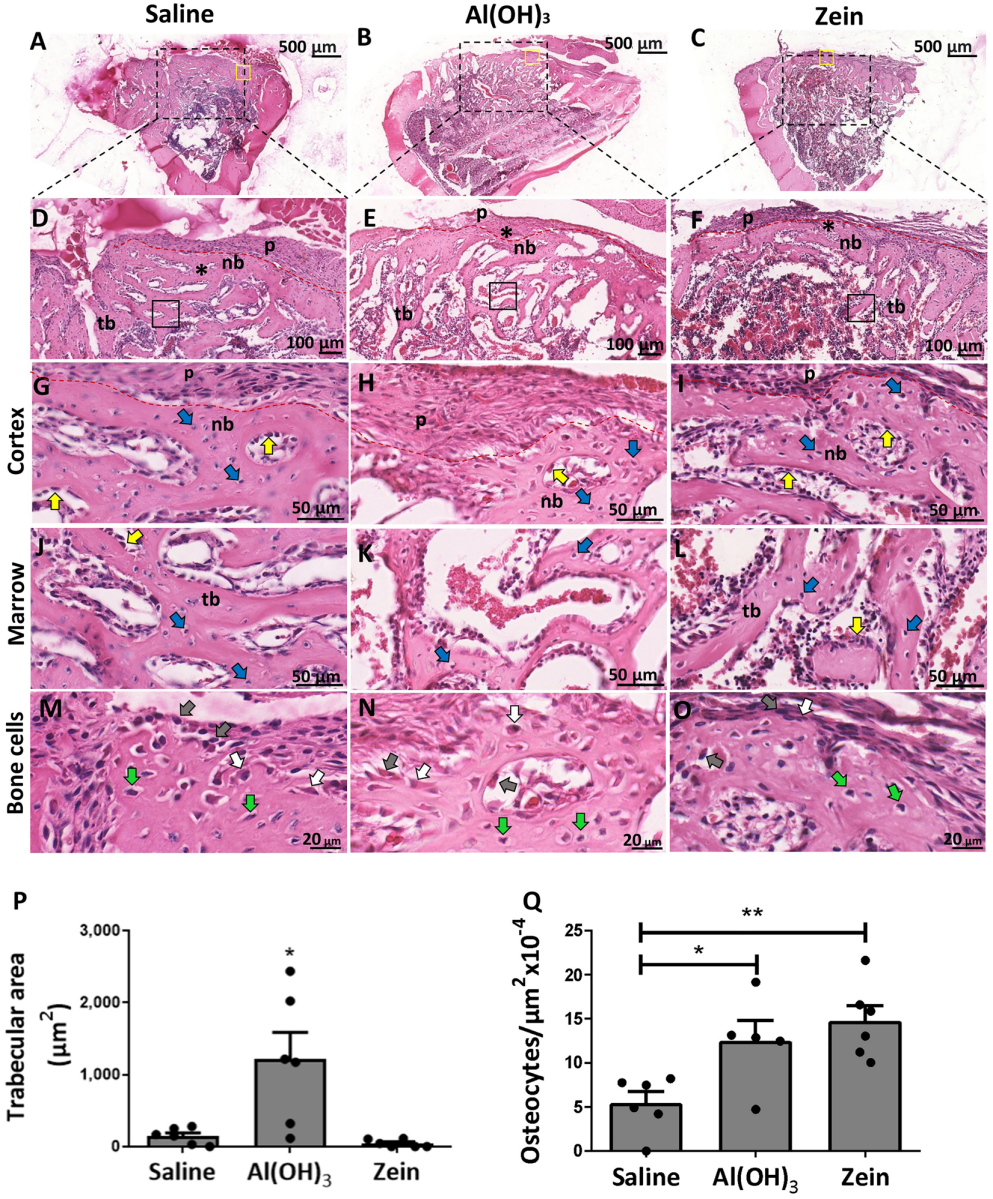
Representative H&E staining images showing the defect region in the rat tibia 14 days post-surgery. (**A**–**C**) Cross-sections of the defect region in the tibia of the studied groups (scale bar = 500 μm, n = 6). (**D**–**F**) The magnification of the defect region is demonstrated by an asterisk (*) and the delimitation of the periosteum by the red dotted line (scale bar = 100 μm, n = 6). (**G**–**I**) Cortical defect bone region demonstrating new bone (nb) formation (scale bar = 50 μm, n = 6). (**J**–**L**) The medullary region was delimited by the square with the presence of trabecular bone (tb) (scale bar = 50 μm, n = 6). Blue arrows indicate osteocytes, and yellow arrows indicate osteoblasts. (**M**–**O**) Corresponding to the stages of bone cell maturation. Gray arrows indicate osteoblasts, white arrows indicate osteoblasts in transition to osteocytes and green arrows indicate mature osteocytes. (**P**) Corresponding quantification of the trabecular bone (tb) area in the medullary region. (**Q**) Corresponding quantification of the osteocytes in the cortical region of the defect. Data represent the mean ± SEM, with **p ≤ 0.01 and ***p ≤ 0.001 for statistical analysis performed between the experimental groups, n = 6.

Fourteen days after the surgery, H&E-stained images revealed the presence of a bone matrix in the cortical region of the defect area associated with connective tissue in the external cortical region in all groups (Fig. 3). However, it is possible to observe SG and AG with a greater area of bone trabeculae in the marrow region compared to ZG (Fig. 3A–F). Periosteal tissue was present in all groups. At greater magnification, it was possible to observe osteoblasts and osteocytes in the cortical and medullary regions in all groups (Fig. 3J–O). In this phase of bone repair, we observed the development of cortical bone in the ZG group, which was better structured than the ones in other groups. Interestingly, the trabecular area was increased in the AG (Fig. 3P), and the number of osteocytes was significantly higher in the region of the defect in the ZG and AG, indicating greater production of bone matrix (Fig. 3Q). These results demonstrate an advance in ZG repair in relation to the other groups.

**Figure 4:**
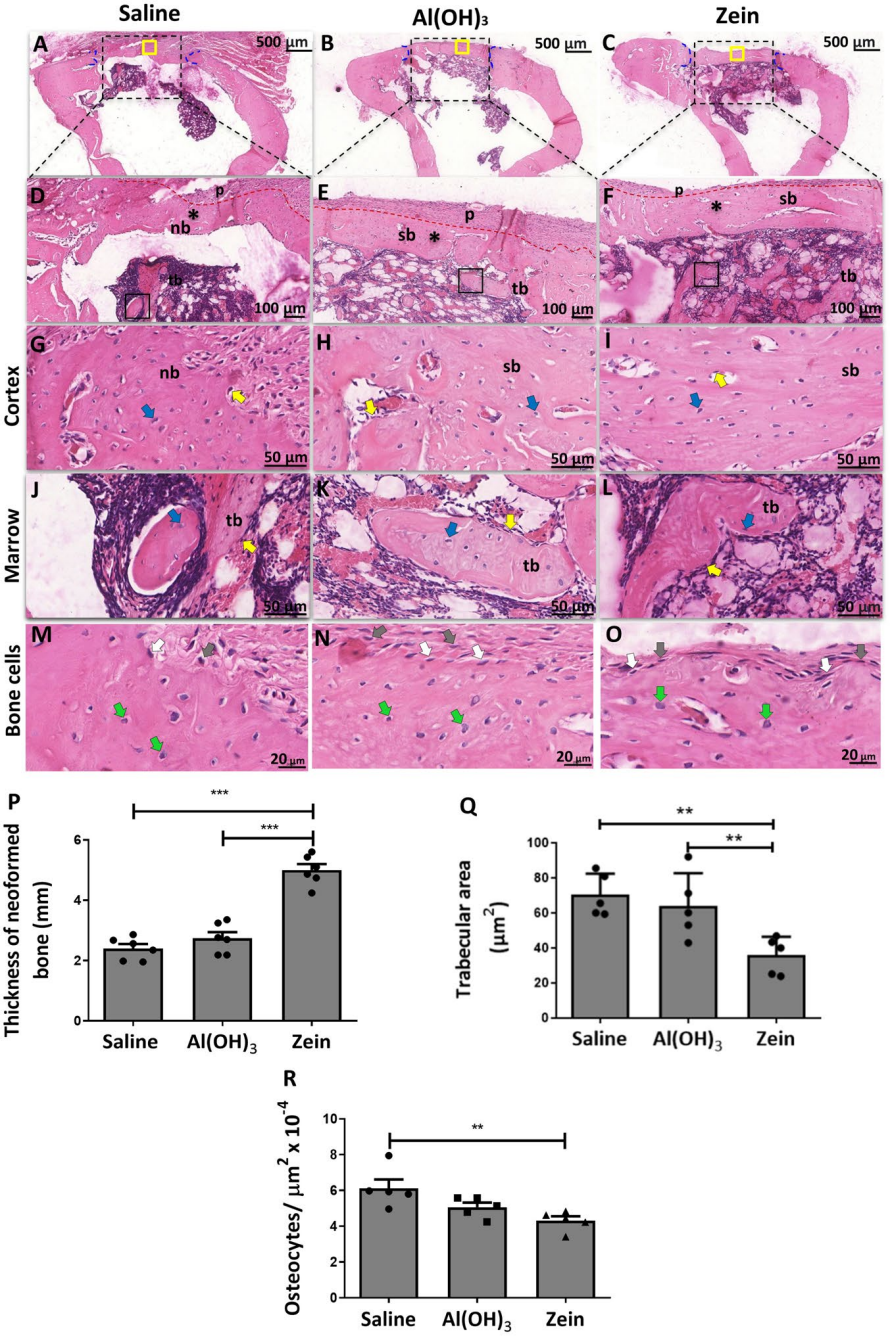
Representative H&E images showing the defect region in the rat tibia 28 days after surgery. (**A**–**C**) Cross-sections of the defect region in the tibia of the studied groups (scale bar = 500 μm, n = 6). (**D**–**F**) The magnification of the defect region is demonstrated by an asterisk (*) and the delimitation of the periosteum by the red dotted line (scale bar = 100 μm, n = 6). (**G**–**I**) Bone region of the cortical defect with the well-delimited presence of osteocytes in the cortical zone and the presence of new bone (nb) and secondary bone (sb) (scale bar = 50 μm, n = 6). (**J**–**L**) The medullary region is bounded by the square with the presence of osteocytes, osteoblasts and trabecular bone (tb) (scale bar = 50 μm, n = 6). Blue arrows indicate osteocytes, and yellow arrows indicate osteoblasts. (**M**–**O**) Corresponding to the stages of maturation of bone cells, gray arrows indicate osteoblasts, white arrows indicate osteoblasts in transition to osteocytes and green arrows indicate mature osteocytes. (**P**) Corresponding to the thickness of the neoformed bone. (**Q**) Corresponding quantification of the trabecular bone (tb) area in the medullary region. (**R**) Corresponding quantification of the osteocytes in the cortical region of the defect. Data represent the mean ± SEM, with **p ≤ 0.01 and ***p ≤ 0.001 for statistical analysis performed between the experimental groups, n = 6.

The findings of the 28-day post-surgery sections point to greater linearity among the experimental groups, with a small layer of periosteal tissue covering the defect area in all groups (Fig. 4A–F). When we analyzed at higher magnification, the cortical zone presented a well-defined presence of osteocytes immersed in the matrix (Fig. 4G–I,M–O) and in the marrow region (Fig. 4J,K,L). However, in the SG, there were characteristics in the bone matrix of tissue compatible with primary bone tissue (nb) (Fig. 4G), mainly due to the irregularity and lack of defined orientation of the collagen fibers. The AG and ZG showed a better arrangement of collagen fibers, in addition to osteoblastic cells, in which the morphology and volume suggested a reduced metabolic activity (Fig. 4N,O). These data might imply the marked presence of secondary bone, demonstrating similarity in the repair process of both groups (Fig. 4H,I,K,L). When the thickness of the neoformed bone was analyzed, the results were greater in the ZG than in the SG and AG (Fig. 4P). On the other hand, the trabecular area in the ZG was smaller than the one in the AG and SG (Fig. 4Q). The number of osteocytes per field was reduced in the ZG compared to the SG (Fig. 4R), and These data together indicated an improved bone repair in ZG when compared to the other experimental groups.

Knowing that the inflammatory process and the injection of tolerated proteins display systemic repercussions, the spleen was evaluated in this period as well. The spleen is a primary lymphoid organ that is directly related to the proliferation and storage of cellular components of the inflammatory process, it was also evaluated by H&E staining. However, no differences were observed in the parameters of the number and area of lymphoid follicles (Supplementary material). This suggests the absence of morphological changes in the spleen caused by immunomodulation induced by *i.p.* injection of zein.

The semi-quantitative analyses of the qualitative morphological parameters on days 7, 14, and 28 were performed by the histological score (Table 2—graphical representation in the Supplementary material).

**Table 2:** Qualitative analysis of predetermined morphological.

	Groups	Bone neoformation in the region of the lesion	Bone trabeculae in the medullary region	Fibrocartilaginous tissue	Inflammatory infiltration
7 days	Saline	+	++	++	+
	Al(OH)_3_	−	++++	++	+++
	Zein	++	+	+	−
14 days	Saline	+	+	++	−
	Al(OH)_3_	+	++	+	−
	Zein	+++	−	+	−
28 days	Saline	+++	+	+	−
	Al(OH)_3_	+++	+	+	−
	Zein	++++	−	+	−

Significant differences were noticed in the variable neoformed bone between the SG and ZG 14 days after the surgery, and we identified differences between the AG and ZG groups at 7 and 14 days, with increase of bone neoformation in the region of the lesion. The trabeculae in the medullary cavity were significant between SG and ZG on days 7 and 14, and between AG and ZG at the same periods. Quantitative differences were absent in fibrocartilaginous tissue at all experimental times among the groups (Supplementary material).

**Figure 5:**
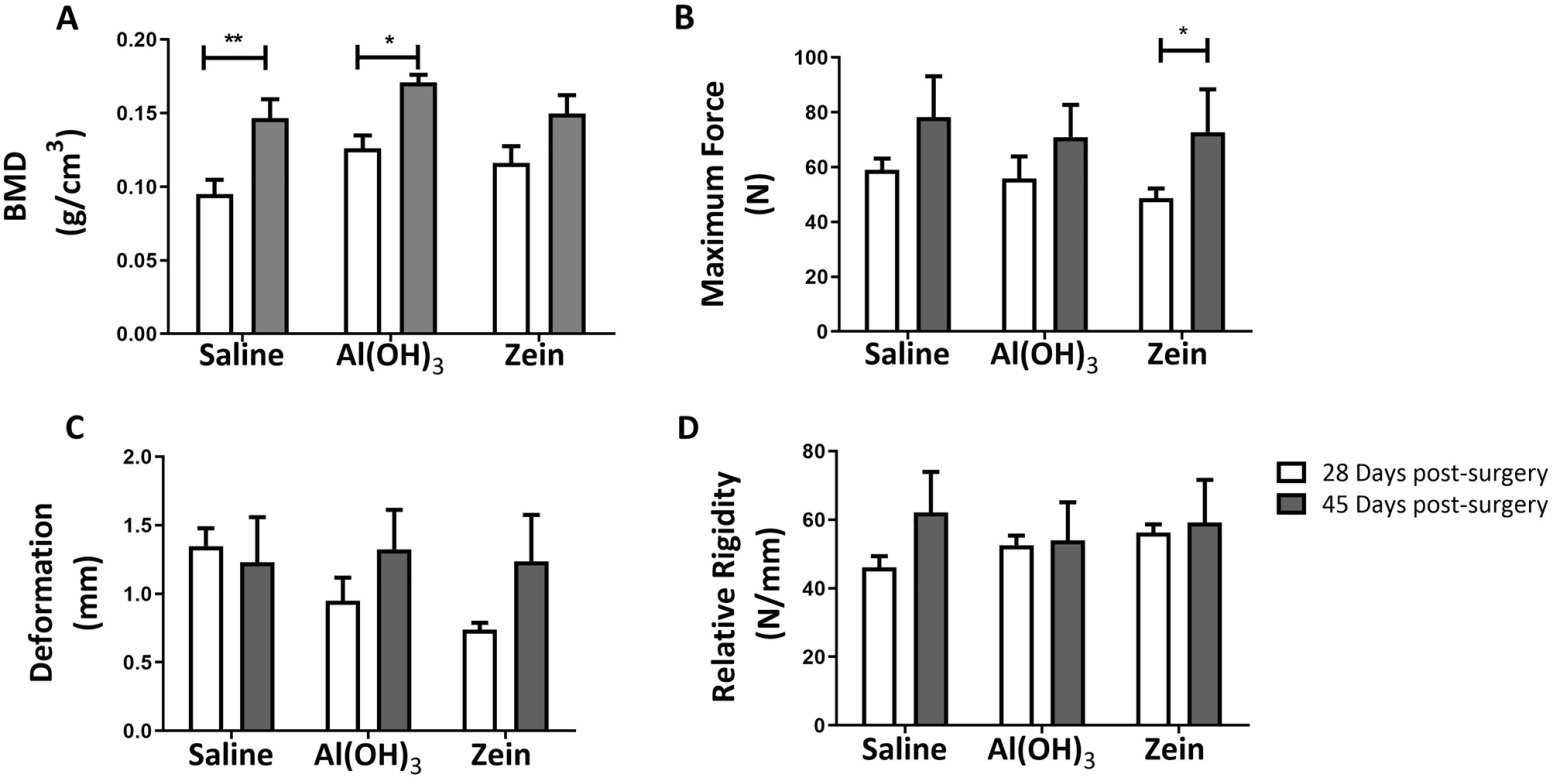
Graphical representations obtained from the BMD and shear tests (**A**) Tibia BMD 28 days after defect. Overall mean of tibia weight: 0.12 g, SD ± 0.01 and tibia BMD 45 days after defect. Overall mean of weight: 0.13 g, SD ± 0.02. (**B**) Graphical representation of the biomechanical parameter maximum force (N). (**C**) Graphical representation of the biomechanical parameter deformation (mm). (**D**) Graphical representation of the biomechanical parameter relative rigidity (N/mm). Data represent the mean ± SEM, with *p ≤ 0.05 and **p ≤ 0.01 for statistical analysis performed between the experimental groups, n = 8.

Aiming at evaluating the bone quality under *i.p*. immunization with dietary protein, biomechanical assays were performed 28 and 45 days after the surgery (Fig. 5). We identified a significant difference in mineral density levels in the SG and AG groups after 28 days compared to the ones after 45 days (Fig. 5A). These findings indicated that animals from both groups did not completely end the process of bone mineralization 28 days after the bone defect process. Surprisingly, the animals in the ZG on day 28 were statistically similar to those in the same group on day 45 (Fig. 5A). These data, associated with H&E staining assays, provided strong evidence that the *i.p.* administration of zein protein in orally tolerated animals can accelerate the bone repair process.

When the results of the mechanical shear tests were compared in the maximum force parameter, the ZG demonstrated a significant increase (Fig. 5B). This result corroborated the BMD analysis in Fig. 5A, and it reinforced that immunization with dietary protein may accelerate the bone repair process and induce more resistant bone formation to external mechanical forces than SG and AG 45 days after the surgery. The parameters of deformation and relative rigidity did not show a difference among the groups (Fig. 5C,D) (Supplementary material).

**Figure 6:**
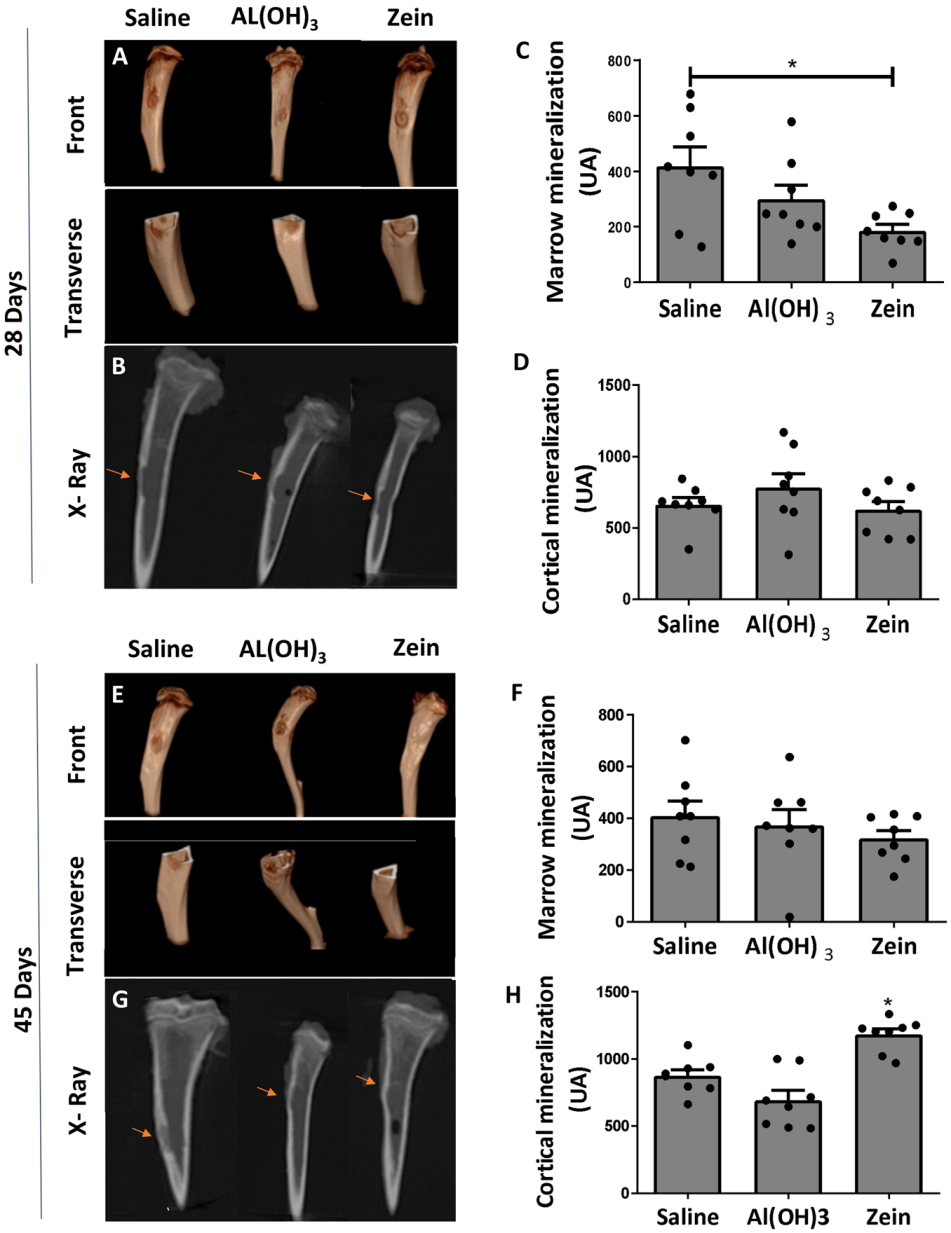
Representative images were obtained by digital computed tomography of the tibia of Wistar rats 28 and 45 days after the surgery. (**A**) Three-dimensional reconstruction of tibiae from 28 days in frontal and transversal views, demonstrating the cortical and medullary region of the bone defect. (**B**) X-ray image obtained by CT scan 28 days post defect, red arrows indicate injured regions. (**C**) Quantitative representation of mineral content levels in the medullary region 28 days post defect. (**D**) Quantitative representation of mineral content levels in the cortical region 28 days post-defect. (**E**) Three-dimensional reconstruction of tibiae from 45 days in frontal and transversal views, demonstrating the cortical and medullary region of the bone defect. (**F**) X-ray image obtained by CT scan 45 days post-defect, red arrows indicate injured regions. (**G**) Quantitative representation of mineral content levels in the medullary region 45 days post-defect. (**H**) Quantitative representation of mineral content levels in the cortical region 45 days post-defect. Data represent the mean ± SEM, with *p ≤ 0.05 for statistical analysis performed between the experimental groups, n = 8.

Once the results demonstrated a significant improvement in histomorphometry and biomechanical analyses in ZG, we decided to evaluate the morphometry and mineralization by computed tomography (CT) 28 and 45 days after the surgery (Fig. 6). The mineralized defect bone was observed to increase significantly in ZG on 3D reconstruction images of CT (Fig. 6A,E). In the graphical representation in Fig. 6D and H, we quantified the mineralization of cortical bone in the defect area. However, in the medullary region of SG, the mineral content is higher in relation to ZG (Fig. 6C,F). This suggested that the presence of bone trabeculae was not reabsorbed in the medullary region in this group yet. Radiographic images revealed that the defect area was not fully mineralized on cortical bone in any of the groups, except in ZG on day 45 (Fig. 6C,D). The results obtained by the analysis of the groups in the period of 45 days indicated higher mineral content in the cortical area of the ZG (Fig. 6B). Radiographs revealed the formation of a thicker layer of mineralized compact bone in the ZG (Fig. 6C) that was similar to cortical bone health from day 45 (Fig. 6D). No difference was found in mineralization levels in the pith region after 45 days (Fig. 6H). These data suggested that oral tolerance after immunization with zein can positively affect bone repair by improving bone quality and accelerating the healing process in non-critical defects in long bones.

## Discussion

Bone fractures are common causes of disability that affect millions of people throughout the world. Normally, bone fracture heals in a predicted sequence of events that initiates immediately after the lesion and may last from weeks to months for complete recovery in humans. The intensity and extension of the inflammatory phase of the bone repair impact the subsequent phases of the healing process and the quality of bone repair. Similarly to bone fracture recovery, the quality of cutaneous wound repair is greatly impacted by its extensive inflammatory phase, which precedes the healing process^26,27^. It is important to develop safe methods to positively interfere with inflammation and improve wound healing. In the present manuscript, we addressed some immunological aspects of tissue repair. More specifically, considering that parenteral injection of an immunologically tolerated protein can influence immune-inflammatory responses to other unrelated antigens. Thus, we investigated if the systemic effects of oral tolerance could modify the bone repair process. Previous works showed that parenteral administration of ovalbumin or zein in tolerant animals displays positive effects on skin wound healing^17^. Previous works have shown that one parenteral injection of proteins (ovalbumin or zein) previously contacted by the oral route, concomitant with or soon after skin lesions, improves cutaneous wound healing in mice^14,17,28–30^. However, to the best of our knowledge, no similar approach to bone repair has been studied so far. In this work, instead of "inducing" oral tolerance to a particular protein at a particular time, zein^14^, a major component of the rat diet in which laboratory animals are already tolerant, was injected to assess its capacity to improve bone healing in rats.

At all time points analyzed, it was possible to notice differences in bone healing after injection of zein. Notably, seven days after the bone defect, there was a reduced inflammatory infiltrate and a higher number of osteocytes in the wound area of the zein group. At this same time, the reduced area of bone trabeculae and increased area of bone neoformation after injection of zein indicated faster bone healing in the experimental group than in the control group. These results were aligned with data from the literature showing a reduced number of inflammatory cells and increased expression of extracellular matrix seven days after skin lesion in mice injected with proteins previously contacted by the oral route^30^. Although prolonged and unregulated inflammation may delay bone healing, it is commonly accepted that physiological inflammation after bone fractures is important for proper bone healing^13^. Accordingly, the use of some anti-inflammatory drugs may negatively impact the quality of bone healing^31^. However, the results demonstrated that the reduction in inflammation was accompanied by adequate bone healing, so the consolidated bone in the zein group showed similar biomechanical properties to the uninjured bone, as attested by histomorphometric, BMD and CT analysis. In addition, there was a bone matrix formation with more osteocytes seven days after the injury in the zein group, showing an improvement in histological repair, which was not evident in the other experimental groups. It is already known that osteoblasts become osteocytes by matrix secretion or by entrapment through adjacent osteoblasts^32^. The speed of matrix deposition can determine the number of embedded osteocytes^33^. The results displayed that seven days after the bone defect an ossification formation was already evident and the number of osteocytes remained high until 14 days after the bone defect. This is exemplified in the osteoid matrix, which is formed much faster and has many embedded osteocytes^34^.

In this work, the osteocytes were identified in the newly formed bone at different stages of the maturation process with a compatible morphology with their function, suggesting appropriate cellular functionality. It was possible to identify osteocytes at 3 stages of the maturation process: (1) embedding osteoid osteocyte, found close to the edge surrounded by a matrix not yet mineralized with fusiform shape and abundant cytoplasm, (2) early mineralizing osteocytes, found involved by little matrix mineralized with abundant cytoplasm and the beginning of the formation of a clear halo around it, characteristic of the gap, (3) mature osteocytes, surrounded by mineralized matrix, nucleus and cytoplasm reduced in an elongated format, presence of a clear halo around it^35^.

Oral tolerance may be correlated with an increase in regulatory T cells (TREG), and it has been shown that, in bone repair, there is an increase in TREG in the bone callus, where the presence of these cells is supposed to play an anti-inflammatory role^36–38^. However, further studies are required to verify the role of these cells in this model of bone repair and their importance in accelerating the deposition of bone matrix. The BMD technique is extremely important for assessing cortical mineralization and is characterized as a powerful resource when diagnosing weaknesses associated with the musculoskeletal system^39^. According to Fonseca et al.^40^, a higher BMD index is associated with increased bone mechanical strength capacity. Our results showed by different techniques a cortical bone formation with adequate morphology, high mineralization levels and better resistance to mechanical stress in the zein group before the other controls group, which characterizes accelerating bone healing.

Analysis of histomorphometric data related to radiographic and biomechanical data clearly showed the positive effect of zein injection, since the zein group reached a good functional outcome of bone repair before the control groups.

Previous studies demonstrated that immunization with tolerated antigens triggers systemic effects capable of modifying various inflammatory phenomena, such as adhesion molecule expression, cytokine production, cell migration and granuloma formation^9,14,16^. This is the first study demonstrating the systemic effects of oral tolerance on bone healing. Oral tolerance is often associated with the activation of T cells and the production of anti-inflammatory cytokines, such as TGF-b and IL-10^10^. Recently, the participation of T cells in wound repair was demonstrated in several organs^41^. It is possible that T cells activated by exposure to orally tolerated proteins reduce inflammation and facilitate bone repair. T cells may improve bone repair through multiple mechanisms that indirectly activate osteoblasts and inhibit osteoclast activation. For example, the production of TGF-b by T cells can increase OPG expression and reduce M-SCF and RANKL expression, minimizing osteoclastogenesis^41^. Thus, the systemic effects of oral tolerance may reduce mineral resorption by osteoclasts. However, other cells and cytokines may be involved in the systemic effects of oral tolerance since not all events triggered by oral tolerance are currently known. The inflammatory phase in bone tissue is highly coordinated by cellular responses and involves the secretion of a series of cytokines and growth factors, and is a determinant of bone formation in subsequent phases^42^. The fact that we observed in our results the presence of neoformed bone in the defect region suggested that the tolerized protein promoted more evident effects in the acute reparative phase and that, in the other phases of bone repair, the effect was attenuated until there was complete tissue repair, as noticed 28 and 45 days after the surgery.

The systemic effects produced by injection of tolerated proteins demonstrated in this and previous studies^9,14,16,17,29^ raised the possibility of their clinical use in multiple situations. Regarding bone healing, low-cost therapies that promote the reduction of inflammation concomitant with an acceleration of bone consolidation are welcome, especially in situations of implants to replace bone pieces, in cases of bone loss due to wearing caused by osteoarthritis or osteoporosis. However, aiming at clinical applications, other studies must be carried out, notably verifying whether injections of zein by other routes of administration display systemic effects on bone repair, as recently demonstrated for cutaneous repair^28^.

## Conclusion

These findings suggested that the injection of zein in previously tolerated animals may improve bone repair, leading to the formation of bone in rats within 7 days post-surgery, with a high number of osteocytes within 28 days post-surgery in the zein group. The zein group bones showed good BMD and mechanical maximum force within 45 days post-surgery. The cortical mineralization was significantly increased in the zein group within 45 days post-surgery. The results indicated that oral tolerance may be a great strategy to bone healing with possibilities for investigations aiming at therapeutic interventions in this field. Further studies on the osteoimmunology involved in the healing process of healthy bones and bones associated with pathologies are required to explore the parenteral application of dietary protein.

## Supplementary Information

Supplementary Information.

## Data Availability

All histological materials, images acquired, and data analyses were performed at the Federal University of São João del Rei, Minas Gerais, Brazil. The datasets used and/or analyzed during the current study are available from the corresponding author upon reasonable request.
