# Supplementary material for: Systemic effects of oral tolerance in bone healing
Source: Sci Rep. 2023 Apr 18;13:6296. doi: 10.1038/s41598-023-33591-4 (PMC10113372; doi:10.1038/s41598-023-33591-4)
Supplement: Supplementary file 1 — Supplementary Information. [file 41598_2023_33591_MOESM1_ESM.pdf]

Supplementary Material

Systemic effects of oral tolerance in bone healing

Bruno Henrique Costa[1], Alisson Kennedy Rezende[1], Lais Costa[1], Gabrielle Fernanda Monteiro Neves[1], Antônio Carlos Shimano[2], Álvaro de Oliveira Penoni[1], Claudia Rocha Carvalho[1,3], Raquel Alves Costa[1], Erika Costa de Alvarenga[1*]

[1]*Departamento de Ciências Naturais, Universidade Federal de São João Del Rei, Praça Dom Helvécio 74, 36301-160, São João del Rei, Minas Gerais, Brazil*

[2]*Departamento de Biomecânica, Medicina e reabilitação do Aparelho Locomotor, Faculdade de Medicina de Ribeirão Preto, Universidade de São Paulo, 14049-900, Ribeirão Preto, São Paulo, Brazil*

[3]*Departamento Morfologia, Instituto de Ciências Biológicas, Universidade Federal de Minas Gerais, 31270-901, Belo Horizonte, Minas Gerais, Brazil*

[*]*Corresponding author: Dr. Erika Costa de Alvarenga (erika.fisio@ufsj.edu.br)*

Contact: +55 (31) 99490-0902

Methods

Histological processing and histomorphometry of spleens

Spleens were fixed with 10% formalin for 48 hours. Subsequently, the spleens were divided into 2 halves by sectioning the center of the transverse plane, which was dehydrated in ethanol and then embedded in paraffin for histological studies. The paraffin blocks were sectioned in serial cross-sections of 5µm. The samples were stained with Hematoxylin and Eosin (H&E). Histological spleen sections were analyzed under a light optical microscope (Olympus BX51). The images were acquired by Moticam 2000 system (2.0 M pixel) and the measurements were performed using ImageJ software (https://imagej.nih.gov/ij). Semiquantitative analyses of spleen sections were performed through the following measures: Area of lymphoid follicle / total area of field examined and Number of lymphoid follicles/field. For the latter, ten random fields were analyzed and then the average number of lymphoid follicles per field was calculated. Finally, the division of the average of follicles by the area of the analyzed field was performed, obtaining a result of the number of follicles / µm2**.**

Supplementary Figures

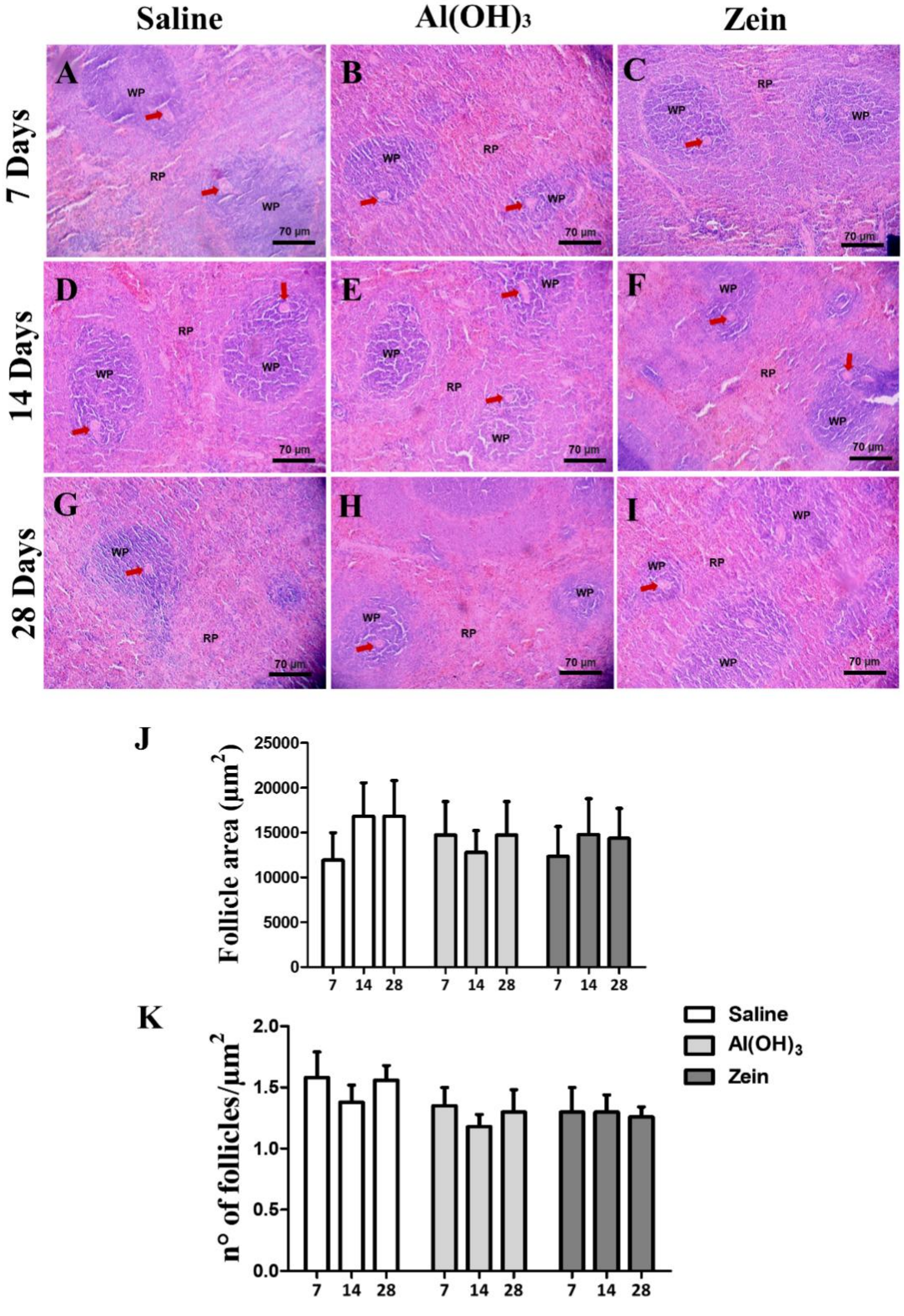

Supplementary figure 1: Representative H&E staining images showing the absence of alterations in the lymphoid follicles on spleens. (**A,B,C**) Group 7 days post-surgery (**D, E, F**) Group 14 days post-surgery (**G, H, I**) Group 28 days post-surgery. White pulps (WP) were indicated in the lymphoid follicles, Red pulp (RP), and central arteriole with red arrows. (All images are with scale bar = 70 μm). (**J**) Corresponding quantification of

lymphoid follicle area. (**K**) Corresponding quantification of number of lymphoid follicles per area. Data represent the mean ± SEM, with p ≤ 0.05 for statistical analysis performed between the experimental groups, n= 6.

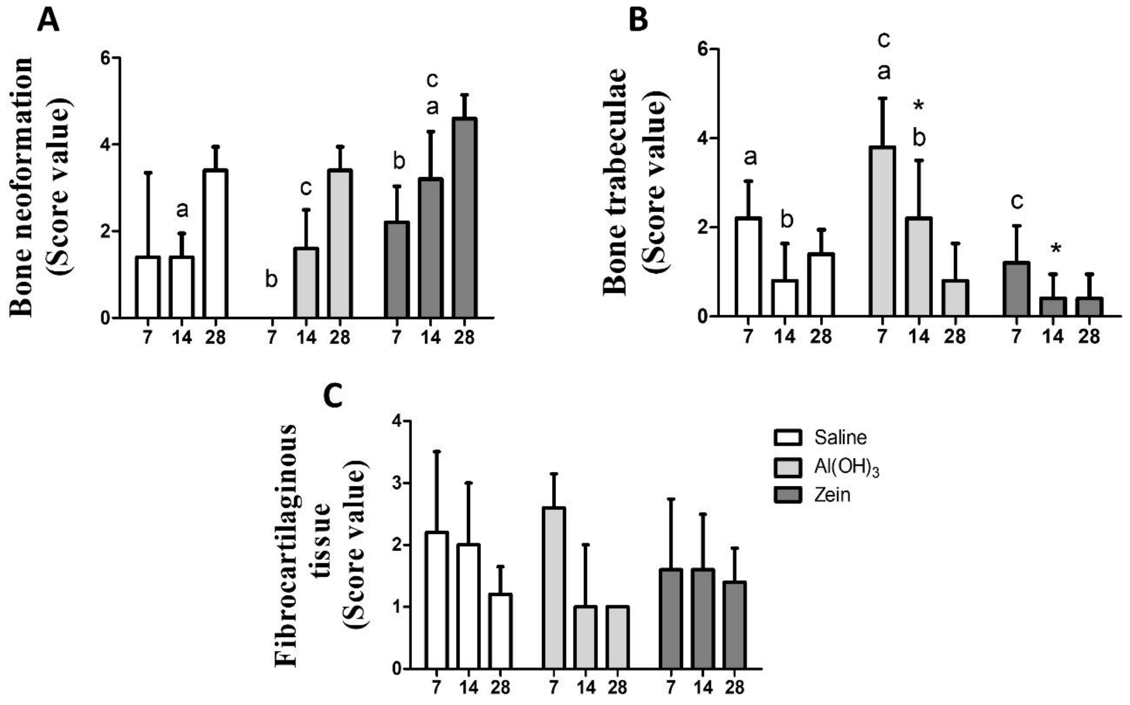

Supplementary figure 2: Graphical representation of semi quantitative parameters. **A)** Neoformed bone (a = p≤ 0.05; b = p≤ 0.01; c = p≤ 0.05). **B)** Trabecular bone (a = p≤ 0.05; b = p≤ 0.05; c = p ≤ 0.001 and * p≤ 0.01) **C)** Fibrocartilaginous tissue. Data represent the mean ± SEM, statistical analysis performed between the experimental groups, n= 6.

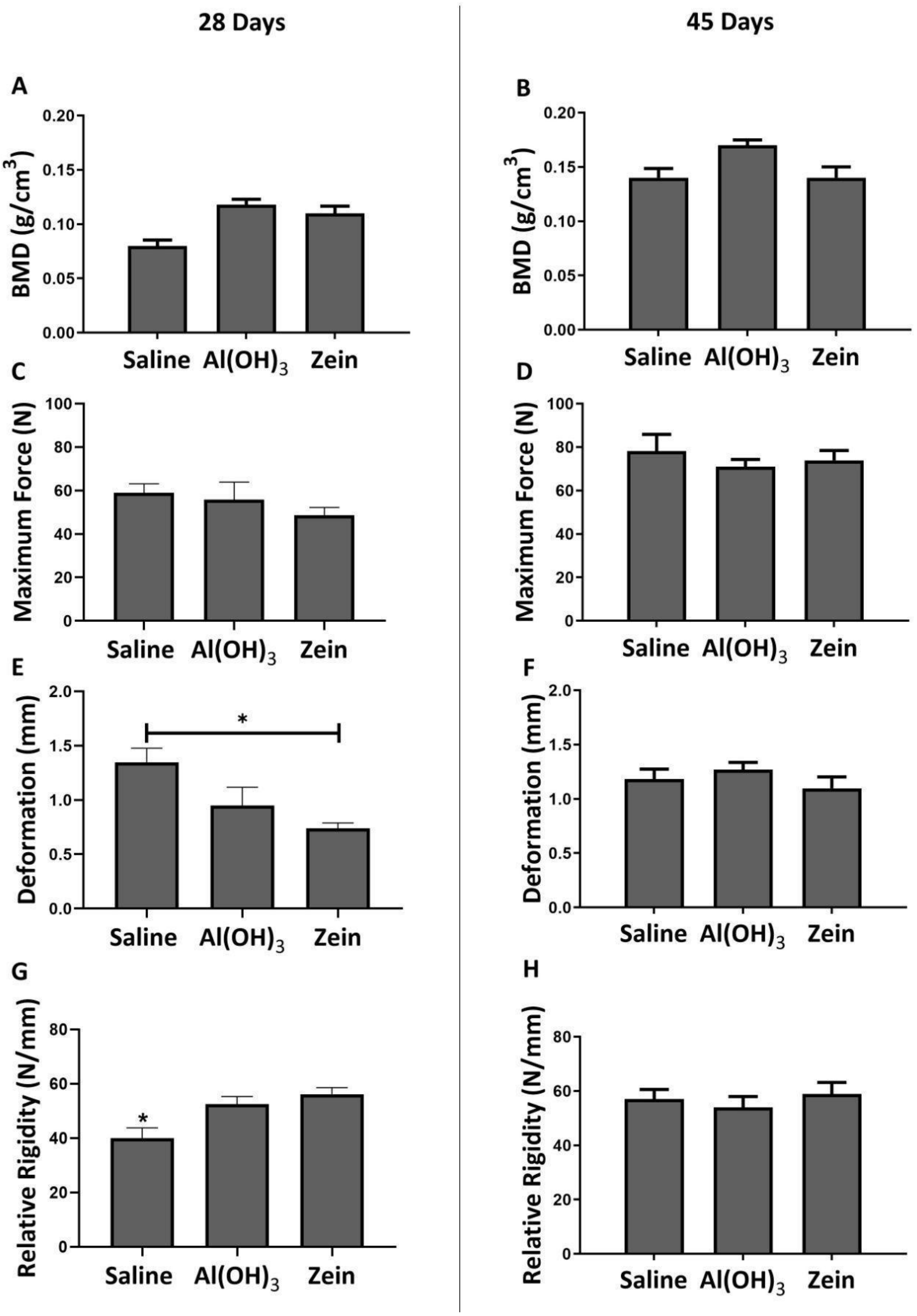

Supplementary figure 3: Graphical representation obtained from the BMD and shear tests at 28 and 45 days post-surgery. **A)** Tibia BMD 28 days after defect. Overall mean of tibia weight: 0.12 g, SD± 0.01 and **B)** tibia BMD 45 days after defect. Overall mean of weight: 0.13 g, SD± 0.02. **C)** and **D)** Graphical representation of the biomechanical

parameter maximum force (N). **E)** and **F)** Graphical representation of the biomechanical parameter deformation (mm). **G)** and **H)** Graphical representation of the biomechanical parameter relative rigidity (N/mm). Data represent the mean ± SEM, with * $p \leq 0.05$ and ** $p \leq 0.01$ for statistical analysis performed between the experimental groups, n=8.
